# Finding answers in lipid profile in COVID-19 patients

**DOI:** 10.1007/s12020-021-02881-0

**Published:** 2021-10-19

**Authors:** M. Sampedro-Nuñez, N. Aguirre-Moreno, L. García-Fraile Fraile, S. Jiménez-Blanco, C. Knott-Torcal, P. Sanz-Martin, G. Fernández-Jiménez, M. Marazuela

**Affiliations:** 1 Department of Endocrinology and Nutrition, Hospital Universitario de la Princesa, 28006 Madrid, Spain; 2 Department of Medicine, Universidad Autónoma de Madrid, 28049 Madrid, Spain; 3 Endocrinology Unit, Instituto de Investigación Sanitaria Princesa, 28006 Madrid, Spain; 4 Department of Endocrinology and Nutrition, Hospital Universitario Rey Juan Carlos, 28933 Madrid, Spain; 5 Department of Internal Medicine, Hospital Universitario de la Princesa, Universidad Autónoma de Madrid, 28006 Madrid, Spain; 6 Department of Clinical Chemistry, Hospital Universitario de la Princesa, Universidad Autónoma de Madrid, Instituto Princesa, 28006 Madrid, Spain; 7 Clinical Information Unit, Hospital Universitario de la Princesa, Universidad Autónoma de Madrid, Instituto Princesa, 28006 Madrid, Spain

**Keywords:** COVID-19, Lipid profile, biomarker, prognosis

## Abstract

**Introduction:**

A small percentage of patients will develop a severe form of COVID-19 caused by SARS-CoV-2 infection. Thus, it is important to predict the potential outcomes identifying early markers of poor prognosis. In this context, we evaluated the association of SARS-CoV-2 infection with lipid abnormalities and their role in prognosis.

**Methods:**

Single-center, retrospective, observational study of COVID-19 patients admitted from March to October 2020. Clinical and laboratory data, comorbidities, and treatments for COVID-19 were evaluated. Main outcomes including intensive care unit (ICU) admission and mortality were analyzed with a multivariable Cox proportional hazards regression model.

**Results:**

We selected 1489 from a total of 2038 consecutive patients with confirmed COVID-19, who had a complete lipid profile before ICU admission. During the follow-up performed in 1109 patients, we observed a decrease in T-c, HDL-c, and LDL-c in 28.6%, 42.9%, and 30.4% of patients, respectively, and an increase in TG in 76.8%. The decrease of both T-c and HDL- c was correlated with a decrease in albumin levels (*r* = 0.39 and *r* = 0.37, respectively). Kaplan–Meier survival curves found an increased ICU admission in patients with lower T-c (HR 0.55, CI 0.36–0.86), HDL-c (HR 0.61, CI 0.45–0.84), and LDL-c (HR 0.85, CI 0.74–0.97). Higher values of T-c (HR 0.45, CI 0.36–0.57), HDL-c (HR 0.66, CI 0.54–0.81), and LDL-c (HR 0.86, CI 0.78–0.94) showed a protective effect on mortality.

**Conclusions:**

Abnormalities in lipid profile are a frequent complication of SARS-CoV-2 infection and might be related to morbidity and mortality.

**Funding:**

Proyectos de Investigación en Salud (FIS) and cofinanced by FEDER.

## Introduction

A new form of coronavirus (Severe Acute Respiratory Syndrome coronavirus 2 or SARS-CoV-2) emerged in December 2019 [1]. The resulting disease was named as COVID-19 and is the biggest global health emergency known in the last century. Spain, and especially Madrid, has been one of the most affected places. Although the majority of COVID-19 patients have mild symptoms and good prognosis [2], some patients rapidly develop a severe disease with acute respiratory syndrome or multiple organ failure [3]. Although, to date, different parameters have been used, there is still no feasible clinical or laboratory index particularly useful to predict the outcomes. Therefore, identification of early severity markers of COVID-19, such as routine tests that can be carried out in practically all clinical settings, will be especially useful for public health purposes.

The prevalence of lipid abnormalities in COVID-19 patients is not well known to date. Some studies have found decreased levels of total cholesterol (T-c), low-density cholesterol (LDL-c), and high-density cholesterol (HDL-c) in COVID-19 patients [4]. A decrease in HDL-c levels and, in some cases, of LDL-c have been associated with a more severe presentation of COVID-19. The association of COVID-19 with triglyceride (TG) levels is not consistent. Several mechanisms have been involved in the origin of dyslipidemia in COVID-19 patients, including the virus itself, the inflammatory pattern or some of the drug therapies used.

Patients with metabolic-associated preconditions such as hypertension, obesity, and diabetes are more susceptible to experience worse outcomes during COVID-19 [5]. In addition, there is recent emerging evidence that vasculopathies and coagulopathies can predict disease severity and mortality. In this regard, as dyslipidemia is associated with metabolic, vascular, and coagulation disorders [5], it could cause more severe symptoms in COVID-19 patients.

The aim of our study was to conduct a thorough assessment and follow-up of the lipid profile in hospitalized patients with COVID-19, including its association with morbidity and mortality. In addition, the association of hipertriglyceridemia (hyper-TG) with different markers of inflammation was analyzed, as well as its potential role in detecting a more severe clinical picture.

## Methods

### Study design and population

**Fig. 1:**
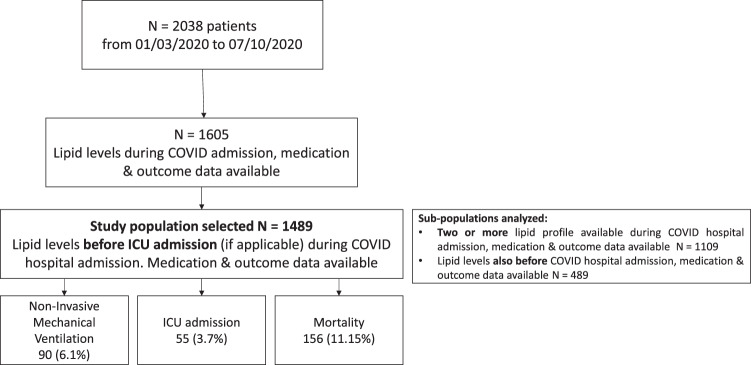
Flow-chart of study population selected for the study

This is a single-center, retrospective, observational study including 1489 from a total of 2038 consecutive patients who had a complete lipid profile before intensive care unit admission (810 [54.4%] male patients, mean age 66.8 ± 16.7 years at admission) with SARS-CoV-2 infection confirmed by RT-PCR performed on nasopharyngeal or oropharyngeal swabs (ThermoFisher Scientific, Waltham, MA USA). All patients were admitted to Hospital Universitario la Princesa due to mild to critical COVID-19 symptoms from March 1^st^ to October 07^th^. 1067 and 422 patients were included in the first (from 1/03 to 14/05/2020) and second wave (from 14/05 to 07/10/2020), respectively. La Princesa Hospital is a 500-bed university referral hospital, including 25 ICU beds, which increased to 60 during the COVID-19 pandemic. A flow-chart of Population selected is shown in Fig. 1.

The highest lipid values prior to their ICU admission were chosen for the analysis in each patient. Lipid levels were measured under fasting conditions, and the normal range was based on cut-off values from healthy controls. LDL-c was calculated using total cholesterol (T-c), HDL-c, and TG levels. Variations in lipid levels were analyzed in 1109 patients who had at least two determinations during hospital admission in the follow-up study. All parameters were analyzed in the same final blood sample. In a cohort of 489 patients who had a lipid profile available before contracting the SARS-CoV-2 infection, both results at admission and during hospitalization were compared.

### Endpoints

Primary endpoint was the impact of lipid profile on ICU admission and mortality. Secondary endpoints were the correlation of lipid levels with other inflammation parameters and the possible interaction of the lipid profile with COVID-19 medications.

### Data collection

Data including demographic and clinical details, as well as laboratory tests and treatments, were obtained from electronic medical records. We collected clinical information including age, gender, previous chronic conditions (smoking habits, diabetes, hypertension, cardiovascular disease, cerebrovascular disease, chronic kidney disease, chronic pulmonary disease, and chronic liver disease). Previous drug therapies for dyslipidemia (statins and fibrates) and diabetes (insulin) were studied. Regarding hospitalization for COVID-19, mode of respiratory support (invasive and non-invasive mechanical ventilation), and treatments received (hydroxichloroquine, antiretrovirals, steroids, and tocilizumab) were also analyzed. Laboratory results included: T-c, LDL-c and HDL-c, TG, routine blood test, erythrocyte sedimentation rate (ESR), fibrinogen, D-dimer, liver function tests (alanine aminotransferase [ALT] and aspartate aminotransferase [AST]), renal function tests (creatinine and blood urea), albumin and prealbumin, lactate dehydrogenase (LDH), fasting blood glucose, glycosylated hemoglobin (HbA1c), high-sensitivity C reactive protein (hsCRP), procalcitonin, ferritin, complement (C3 and C4), immunoglobulins (IgA, IgG, and Ig M), and interleukin-6 (IL-6). Furthermore, laboratory results were included on several time points. Biochemical and lipid parameters were classified according to the day of symptoms in values before COVID-19 initial symptoms (pre-COVID-19), from initial symptoms to day 6 (initial disease), from day 7 to day 14 of symptoms (progression) or posterior to day 14 of symptoms (follow-up). Other outcomes measured included non-invasive (e.g., BiPAP) and invasive mechanical ventilation to maintain an oxygen saturation of ≥90, intensive care unit (ICU) admission, and in-hospital death.

### Statistical analysis

Quantitative variables are expressed as the median and interquartile range (boxplots), while qualitative variables are presented as relative percentages of samples (histograms), included in contingency tables. Fisher’s exact test was used to compare qualitative variables. The unpaired, two-tailed, Student’s *t* test was performed to compare two independent groups, as well as the paired Student’s *t* test to analyze two related samples or their non-parametric variant if sample distribution was abnormal (Wilcoxon test). We also used false discovery rate (FDR) correction when conducting multiple comparisons. Two factor ANOVA was performed to evaluate the effect of medication on lipid profile variation during COVID-19 admission. netCoin R package [6] was used to create a network coincidence graph and evaluate the pattern between comorbidities during COVID-19 admission. Furthermore, variation between two time points for each biochemical parameter was calculated and with these values, we performed the Spearman’s rho analysis to find correlations between blood markers changes on time (positive rho values indicates variation in the same direction while negative rho values indicate variation on opposite direction between two biochemical parameters. A correlation map was conducted with all these Spearman’s rho values using R package corrplot (available from: https://github.com/taiyun/corrplot), and all its variables were reordered using hierarchical cluster method. Finally, we used Cox regression analyses to analyze the effect of the different biomarkers on the survival rate and ICU-admission in this cohort of patients. Stata version 12.0 for Windows and R version 3.3.2 were used for all the statistical analysis. *P*-values < 0.05 were deemed statistically significant.

### Ethics

Ethics approval was granted by the local Research Ethics Committee in accordance with the ethical principles established in the Declaration of Helsinki.

## Results

### COVID-19 patients have a decrease in cholesterol and its subfractions as well as an increase in triglycerides

Figure 1 illustrates a flowchart of patient recruitment. The study population with a valid lipid profile before ICU admission (if applicable) included a total of 1489 patients. Median age was 66.8 ± 16.7, and 45.6% were females and 54.4% were men. Out of the total, 1126 patients (75.6%) had one or more chronic illnesses (Table 1). The most common comorbidities were hypertension (45.9%), chronic pulmonary disease (20.8%), diabetes (20.6%), cardiovascular disease (17.0%), and cerebrovascular disease (15.9%). The median length of hospital stay was 8.2 days (p25-p75 5.8 to 12.9). 156 patients (11.2%) died, 90 patients (6.1%) required non-invasive mechanical ventilation, and 55 (3.7%) required ICU admission (Fig. 1). The average time in the ICU was 5 days (p25-p75 3 to 10). In the cohort of 489 patients who had lipid profile done on the previous year before their COVID-19-related admission, 164 (34.1%) had an abnormal lipid profile. Regarding pharmacological treatments prior to admission, 437 (29.4%) patients were under statin therapy, 33 (2.25%) were receiving fibrates, and 76 (5.1%) were on insulin treatment (Table 1). Median values of T-c, HDL-c, LDL-c, and TG during admission of the 1489 patients were 202 mg/dL (p25-p75 = 170–232), 45 (p25-p75 = 34–59), 129.5 (p25-p75 = 108–148), and 162 mg/dL (p25-75 = 118–230), respectively (Table 2).

**Table 1:** Characteristics of patients with COVID-19 by mortality and severity status

	Total (*n* = 1489)	Survivor (*n* = 1333)	Non-Survivor (*n* = 156)		Mild disease (*n* = 1362)	Severe disease (*n* = 127)	
	Number (%)	Number (%)	Number (%)	*P* value	Number (%)	Number (%)	*P* value
Age, mean (± SD), years	66.83 ± 16.74	65.24 ± 16.47	80.35 ± 12.42	<0.001	66.96 ± 17.03	65.34 ± 13.21	0.296
Male (*n* valid = 1489)	810 (54.40)	722 (54.16)	88 (56.41)	0.611	727 (53.38)	83 (65.35)	0.012
Female	679 (45.60)	611 (45.84)	68 (43.59)		635 (46.62)	44 (34.65)	
**Comorbidities**	1126 (75.62)	980 (73.52)	146 (93.59)	<0.001	1023 (75.11)	103 (81.10)	0.160
Non-Smoker (*n* valid = 1070)	755/1070 (70.56)	696/963 (72.27)	59/107 (55.14)	0.003	701/976 (71.82)	54/94 (57.45)	0.009
Smoker	65/1070 (6.07)	58/963 (6.02)	7/107 (6.54)		59/976 (6.05)	6/94 (6.38)	
Ex-smoker	250/1070 (23.36)	209/963 (21.70)	41/107 (38.32)		216/976 (22.13)	34/94 (36.17)	
Hypertension (*n* valid = 1489)	684 (45.94)	581 (43.59)	103 (66.03)	<0.001	624 (45.81)	60 (47.24)	0.780
Hypercholesterolemia (*n* valid = 1489)	566 (38.01)	492 (36.91)	74 (47.44)	0.011	508 (37.30)	58 (45.67)	0.069
Hypertriglyceridemia (*n* valid = 1489)	72 (4.84)	62 (4.65)	10 (6.41)	0.324	60 (4.41)	12 (9.45)	0.017
Diabetes (*n* valid = 1489)	307 (20.62)	252 (18.90)	55 (35.26)	<0.001	271 (19.90)	36 (28.35)	0.029
Cardiovascular disease (*n* valid = 1488)	253/1488 (17.00)	205/1332 (15.39)	48/156 (30.77)	<0.001	237/1362 (17.40)	16/126 (12.70)	0.215
Cerebrovascular disease (*n* valid = 1488)	237/1488 (15.93)	190/1332 (14.26)	47/156 (30.13)	<0.001	226/1361 (16.61)	11/127 (8.66)	0.022
Chronic pulmonary disease (*n* valid = 1489)	309 (20.75)	262 (19.65)	47 (30.13)	0.003	276 (20.26)	33 (25.98)	0.137
Chronic renal disease (*n* valid = 1489)	113 (7.59)	90 (6.75)	23 (14.74)	0.001	102 (7.49)	11 (8.66)	0.600
Chronic liver disease (*n* valid = 1488)	80/1488 (5.38)	71/1332 (5.33)	9/156 (5.77)	0.851	75/1361 (5.51)	5/127 (3.94)	0.679
Insulin treatment (*n* valid = 1489)	76 (5.10)	60 (4.5)	16 (10.26)	0.006	69 (5.07)	7 (5.51)	0.832
Statin treatment (*n* valid = 1489)	437 (29.35)	377 (28.28)	60 (38.46)	0.009	389 (28.56)	48 (37.80)	0.032
Fibrate treatment (*n* valid = 1489)	33 (2.22)	26 (1.95)	7 (4.49)	0.075	26 (1.91)	7 (5.51)	0.018°

Values are classified by mortality status and severity at the end of follow-up

Patients were considered severe cases if advance ventilation (VMNI) or ICU admission were needed during admission

**Table 2:** Biochemical peak/valley values of patients with COVID-19

		Survivor (*n* = 1333)	Non-survivor (*n* = 156)		Mild disease (*n* = 1362)	Severe disease (*n* = 127)	
	Normal range	Median (IQR)	Number (IQR)	*P* value	Median (IQR)	Number (IQR)	*P* value
**Peak red blood cells (*N.*** x 1000 cells)	3.80–4.80	4.93 (4.57–5.27)	4.73 (4.25–5.04)	0.000108	4.9 (4.52–5.25)	4.96 (4.73–5.34)	0.047400
**Peak hemoglobin**	12.0–15.0	14.6 (13.5–15.6)	13.7 (12.55–15)	0.000016	14.6 (13.4–15.6)	14.8 (13.8–15.9)	0.040067
**Peak hematocrit**	36.0–46.0	44.3 (41.4–47)	42.6 (38.55–45.95)	0.000461	43.9 (41.12–46.8)	44.6 (42.4–47.6)	0.047400
**Peak platelets (*N.*** x 1000 cells)	150–450	343 (280–441)	253 (194.5–372.5)	0.000000	330 (268–428.75)	387 (295–515)	0.000533
**Peak total leukocytes cells (*N.*** x 1000 cells)	4.00–10.00	10.07 (7.54–13.35)	13.26 (9.19–19.54)	0.000000	9.9 (7.38–13.13)	16.16 (12.25–23.2)	0.000000
**Peak neutrophils (*N.*** x 1000 cells)	1.50–8.00	8.02 (5.27–11.1)	11.19 (7.73–17.24)	0.000000	7.87 (5.16–10.84)	13.66 (10.73–19.89)	0.000000
**Peak lymphocytes (*N.*** x 1000 cells)	1.00–4.00	2.2 (1.69–2.83)	1.35 (0.81–1.89)	0.000000	2.13 (1.55–2.76)	2.14 (1.53–2.83)	0.988859
**Peak monocytes (*N.*** x 1000 cells)	0.20–0.80	0.77 (0.61–1)	0.71 (0.44–1.1)	0.090782	0.75 (0.58–0.98)	1.04 (0.8–1.37)	0.000000
**Peak eosinophils (*N.*** x 1000 cells)	0.20–0.50	0.2 (0.12–0.33)	0.09 (0.02–0.23)	0.000000	0.19 (0.11–0.31)	0.27 (0.13–0.56)	0.000857
**Peak basophils (*N.*** x 1000 cells)	0.00–0.20	0.05 (0.03–0.07)	0.03 (0.02–0.06)	0.000169	0.05 (0.03–0.07)	0.07 (0.04–0.11)	0.000002
**Peak ESR**	0.0–25.00	45 (25–70)	69 (51–83)	0.000004	47 (26–72)	50.5 (28–74)	0.542465
**Peak D dimer**	0.15–0.50	1.04 (0.59–2.18)	5.66 (1.68–18.45)	0.000000	1.05 (0.6–2.19)	5.14 (1.77–18.2)	0.000000
**Peak fibrinogen**	150–400	765 (663.75–872.25)	779.5 (667–955.75)	0.207711	758.5 (652.75–863.25)	882 (763.25–1006.5)	0.000000
**Peak T-c**	150–200	205 (174–235)	165.5 (138–195.5)	0.000000	199 (169–230)	229 (184.25–263.75)	0.000066
**Peak TG**	50–200	162 (118–228)	163.5 (119–247)	0.557804	157 (114–214)	283 (186.5–403)	0.000000
**Valley HDL-c**	≥65	46 (35–60)	31 (22.5–46.5)	0.000016	47 (36–60)	34 (24–46)	0.000000
**Valley LDL-c**	≤130	130 (110–149)	106 (66–133)	0.003386	130 (108–148.25)	128.5 (107.25–146)	0.751943
**Peak random blood glucose**	74–106	142 (118–180)	173 (140–229.5)	0.000000	141 (118–177)	197 (158–288)	0.000000
**Peak HbA1c**	4.0–5.7	6 (5.7–6.7)	6.8 (5.85–7.4)	0.130892	6 (5.7–6.7)	6.3 (5.8–6.9)	0.316584
**Valley albumin**	3.5–5.2	3.5 (3.2–3.9)	3.2 (2.8–3.5)	0.000000	3.5 (3.2–3.9)	3 (2.8–3.4)	0.000000
**Valley prealbumin**	20.00–40.00	20.86 (14.62–29.11)	13 (8.22–34.18)	0.573891	19.14 (12.67–26.13)	26.15 (17.12–36.88)	0.002552
**Peak creatinine**	0.50–0.90	0.99 (0.82–1.21)	1.27 (0.94–2.01)	0.000000	1 (0.83–1.23)	1.09 (0.85–1.5)	0.012779
**Peak blood urea**	16.6–48.5	47 (36–65)	90 (63–132)	0.000000	47 (36–66)	74 (55–98)	0.000000
**Peak AST**	4–32	50 (34–79)	62 (41–97)	0.003386	49 (33–77)	77 (53–116)	0.000000
**Peak ALT**	5–33	59 (34–105)	44 (25–107)	0.014899	54 (32–97)	119 (73–203)	0.000000
**Peak ferritin**	15–150	956 (442.5–1758.5)	1525 (889–2848)	0.000016	945 (437.25–1752.25)	1655 (1119.5–2702.5)	0.000000
**Peak IL-6**		14.81 (3.69–44.24)	68.5 (21–197.89)	0.000000	13.28 (3.65–37.6)	87 (21.5–336.54)	0.000000
**Peak LDH**	135–214	325 (264–411.75)	530 (422–752)	0.000000	324 (263.25–412)	563 (435–727)	0.000000
**Peak hsRCP**	0.00–0.50	9.77 (5.12–16.97)	19.32 (12.25–30.45)	0.000000	9.86 (5.12–16.85)	20.11 (11.57–30.34)	0.000000
**Peak IgG**	800–1600	1060 (864.5–1240)	953 (768.5–1165)	0.057210	1055 (865.75–1250)	1010 (803.25–1180)	0.066501
**Peak IgA**	100–300	250 (174.5–334)	275 (192.5–352.5)	0.207711	260 (178–339)	220 (169–309.5)	0.099928
**Peak IgM**	80–250	97.5 (73.97–141)	102 (59–140)	0.573891	98 (73.55–141)	94.9 (60.65–140.75)	0.640211
**Peak C3**	80–140	125 (109–143)	108 (102–126)	0.006473	124.5 (107.75–143)	124.5 (108.25–137.75)	0.714261
**Peak C4**	12–33	29 (21.8–35.6)	27.4 (23.4–36.1)	0.573891	29.15 (21.68–35.75)	28.35 (23.15–34.57)	0.751943
**Peak procalcitonin**	0.05–0.09	0.15 (0.09–0.32)	0.48 (0.19–1.48)	0.000000	0.15 (0.08–0.31)	0.38 (0.19–1.43)	0.000000

Values are classified by mortality status and severity at the end of follow-up

Patients were considered severe cases if advance ventilation (VMNI) or ICU admission were needed during admission

Patients were classified regarding mortality between survivors and non-survivors, and regarding ICU admission. The non-survivor group of patients were more likely to be older individuals with a higher prevalence of comorbidities. A correlation was found between survival rates and several variables including hypertension (*p* < 0.001), previous history of smoking (*p* = 0.003), hypercholesterolemia (*p* = 0.011), diabetes (*p* < 0.001), cardiovascular disease (*p* < 0.001), cerebrovascular disease (*p* < 0.001), chronic pulmonary disease (*p* = 0.003) and chronic renal disease (*p* < 0.001). Non-survivors group had more insulin treatment (*P* = 0.006) and statin therapy (*p* = 0.009). Critically ill patients also had a higher prevalence of statin (38% vs 29%) and fibrate use (5% vs 2%) than those in the mild disease group (Table 1). However, when more variables including comorbidities, age, and sex were added in the multivariable analysis, the effect of statins on survival was not significant (neither a risk nor a protective factor).

### Relation of lipid profile with comorbidities and previous therapies

**Fig. 2:**
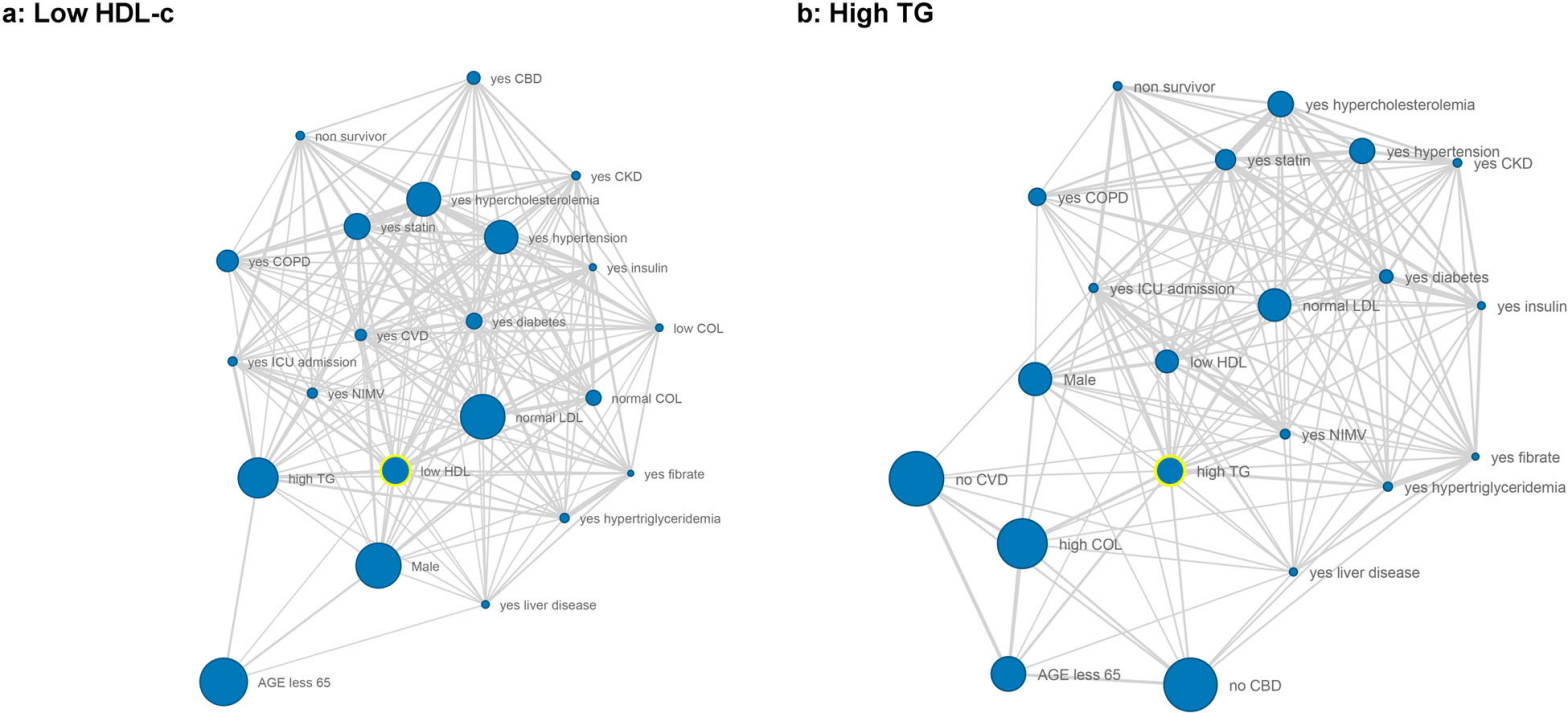
Network graph of coincidences within different lipid profiles during COVID-19. Patients with low HDL-c (max HDL < 40 mg/dL, *n* = 201) or high TG (max TG value ≥200 mg/dL, *n* = 494) were filtered in a network analysis to find relationships with other clinical parameters. Nodes for general characteristics (age, BMI, previous comorbidities) were included. Node size correlates to the prevalence of that variable and a shorter distance between the nodes indicates a greater co-occurrence of features. Statistical weight and width of the links between nodes were calculated by Haberman distance

Network coincidence analysis was used to evaluate the relationship between lipid profile abnormalities, comorbidities, and previous therapies in COVID-19. Coincidence analysis detects what qualitative variables tend to occur together [6]. In Fig. 2, node size correlates to the prevalence of that variable and a shorter distance between the nodes indicates a greater co-occurrence of features. Interestingly, patients with low HDL-c values were correlated with younger male patients with multiple comorbidities (hypertension, diabetes, hypercholesterolemia, COPD, CKD, and liver disease). These patients who needed more non-mechanical invasive ventilation (NVMI), were more likely to be admitted to the ICU and/or were non-survivors. Interestingly, high TG related network had similar topography to reduced HDL-c when it was analyzed (Fig. 2).

### Effect of different therapies used during hospitalization on lipid profile

Regarding medical therapy during hospital admission, of the total of 1109 patients with follow-up, 754 (68.0%) received hydroxychloroquine, 520 (46.9%) LPV/r, 678 methylprednisolone (61.4%), 196 (17.7%) dexamethasone, and 167 (15.1%) tocilizumab (Table 3). Tocilizumab was administered in cases of clinical suspicion of a severe hyperinflammatory state. The average time of use of these medications was 5 days for hydroxychloroquine, as well as for LPV/r, and methyldprednisone, 7 days for dexamethasone, and 1 day for tocilizumab. No significant variations were found between the different therapies used except for tocilizumab (Table 3). Interestingly, patients treated with tocilizumab had a higher increment of TG than those who did not receive this treatment (interaction *p*-value < 0.05).

**Table 3:** Two-factor ANOVA for evaluation of COVID-19 medication on lipid profile variation

	Hydroxychloroquine			
	Time^a^	No	Yes	Interaction *P*-value^b^
**T-c (mg/dL)**	**Pre**	163.62 ± 30.95	169.98 ± 46.20	0.44198
	**Pos**	161.78 ± 35.01	157.89 ± 40.99	
**HDL-c (mg/dL)**	**Pre**	56.20 ± 6.06	52.61 ± 20.81	0.43691
	**Pos**	37.27 ± 11.65	41.57 ± 17.03	
**LDL-c (mg/dL)**	**Pre**	68.80 ± 31.09	113.95 ± 37.03	0.05
	**Pos**	119.78 ± 36.99	117.63 ± 41.59	
**TG (mg/dL)**	**Pre**	125.79 ± 55.85	166.77 ± 148.84	0.0514
	**Pos**	161.97 ± 87.98	167.83 ± 99.74	
	**LPV/r**			
	**Time**^a^	**No**	**Yes**	**Interaction** ***P*-value**^b^
**T-c (mg/dL)**	**Pre**	163.36 ± 43.02	173.82 ± 45.66	0.6885
	**Pos**	153.36 ± 41.99	160.59 ± 39.71	
**HDL-c (mg/dL)**	**Pre**	52.50 ± 14.54	53.55 ± 23.10	0.624159
	**Pos**	42.64 ± 17.12	40.27 ± 16.23	
**LDL-c (mg/dL)**	**Pre**	108.50 ± 37.82	106.37 ± 41.43	0.496
	**Pos**	127.48 ± 38.45	113.57 ± 41.49	
**TG (mg/dL)**	**Pre**	135.17 ± 70.62	173.44 ± 175.62	0.1368
	**Pos**	160.68 ± 82.42	171.49 ± 108.50	
	**Methylprednisolone**			
	**Time**^a^	**No**	**Yes**	**Interaction** ***P*-value**^b^
**T-c (mg/dL)**	**Pre**	175.62 ± 45.52	166.84 ± 44.33	0.32231
	**Pos**	158.23 ± 40.01	158.12 ± 40.92	
**HDL-c (mg/dL)**	**Pre**	59.17 ± 22.57	51.19 ± 18.47	0.106861
	**Pos**	37.50 ± 15.64	42.27 ± 16.69	
**LDL-c (mg/dL)**	**Pre**	125.33 ± 32.54	100.82 ± 40.06	0.0815
	**Pos**	112.07 ± 45.69	120.36 ± 38.79	
**TG (mg/dL)**	**Pre**	129.97 ± 57.36	158.67 ± 140.82	0.4884
	**Pos**	155.61 ± 60.64	171.70 ± 110.66	
	**Dexamethasone**			
	**Time**^a^	**No**	**Yes**	**Interaction** ***P*-value**^b^
**T-c (mg/dL)**	**Pre**	170.09 ± 46.01	162.74 ± 32.92	0.7449
	**Pos**	158.90 ± 40.60	147.16 ± 39.40	
**HDL-c (mg/dL)**	**Pre**	53.97 ± 20.02	45.50 ± 13.92	0.441664
	**Pos**	40.99 ± 16.75	41.67 ± 13.04	
**LDL-c (mg/dL)**	**Pre**	105.74 ± 40.04	116.40 ± 37.35	0.814
	**Pos**	116.95 ± 41.24	134.38 ± 32.51	
**TG (mg/dL)**	**Pre**	150.12 ± 128.51	139.08 ± 60.39	0.7187
	**Pos**	166.70 ± 100.03	163.31 ± 81.15	
	**Tocilizumab**			
	**Time**^a^	**No**	**Yes**	**Interaction** ***P*-value**^b^
**T-c (mg/dL)**	**Pre**	169.25 ± 44.25	169.30 ± 47.72	0.59407
	**Pos**	157.09 ± 41.00	162.72 ± 38.67	
**HDL-c (mg/dL)**	**Pre**	53.19 ± 20.91	52.50 ± 10.01	0.54881
	**Pos**	42.84 ± 16.58	36.91 ± 15.74	
**LDL-c (mg/dL)**	**Pre**	112.77 ± 39.85	81.83 ± 25.90	0.234
	**Pos**	119.29 ± 43.34	113.56 ± 32.44	
**TG (mg/dL)**	**Pre**	149.53 ± 121.54	131.50 ± 61.20	0.0477^a^
	**Pos**	160.87 ± 85.36	198.29 ± 143.83	

^a^Pretreatment (pre) were from -7 to 0 days before the treatment was initiated. Post-treatment biochemical results were from day +1 to day +14 after the initial dose of each treatment

^b^Interaction *P*-value < 0.05

### Variations of lipid profile, albumin, and inflammatory markers in relation with severity of the disease

When associations between mortality and lipid profile, as well as among mortality and hepatic and inflammatory markers were examined, the non-survivor group had significantly different baseline profiles than the survivor group (Table 2). Also, the non-survivor group had lower levels of T-c (*p* < 0.0001), HDL-c (*p* < 0.0001), and LDL-c (*p* < 0.0001). No differences were observed in TG. Similarly, patients with a severe presentation of the disease had lower HDL-c levels (*p* < 0.0001) but higher levels of TG than those with a mild disease (*p* < 0.0001).

In addition, other parameters which had been previously reported as markers of severity in SARS-CoV-2 infection, such as leukocytosis (*p* < 0.0001), as well as an increase in ESR (*p* < 0.0001), D dimer (*p* < 0.0001), hsCRP (*p* = 0.004), procalcitonin (*p* < 0.0001), aspartate aminotransferase (*p* < 0.005), lactate dehydrogenase (*p* < 0.0001), and ferritin (*p* < 0.0001), were higher in the non-survival group (Table 2).

**Fig. 3:**
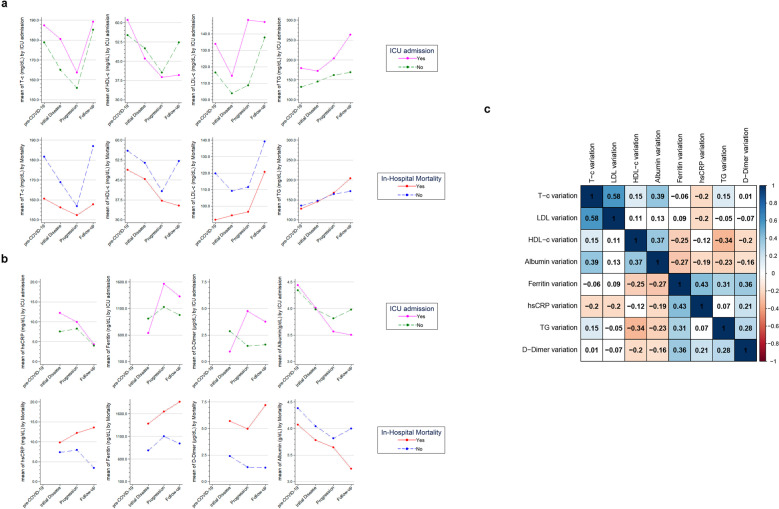
Lipid and inflammatory profile evolution before and during COVID-19 admission. Total cholesterol (T-c), cholesterol fractions (HDL-c and LDL-c), triglycerides (TG), and inflammatory markers (hsCRP, Ferritin, D-dimer and albumin) were measured in venous blood samples of COVID-19 patients. Comparison of patients with and without ICU-admission, as well as the comparison of patients according to mortality rates (no/yes) are also displayed. Line graphs represent trends of mean blood samples values before COVID-19 initial symptoms (**pre-COVID-19**, *n* = 489), from initial symptoms to day 6 (**initial disease**, *n* = 337), from day 7 to day 14 of symptoms (**progression**, *n* = 801) or posterior to day 14 of symptoms (**follow-up**, *n* = 1071) for: **a** lipid profile markers, **b** different inflammatory markers, and **c** correlation of lipid profile variation with other laboratory parameters in COVID-19 patients. Variation between two time points for each biochemical parameter was calculated and with these values, we performed the Spearman’s rho analysis to find correlations between blood markers changes on time (positive rho values indicates variation in the same direction while negative rho values indicate variation on opposite direction between two biochemical parameters). Values represent the Spearman’s rank correlation coefficient, rho (ρ) between the variations in each laboratory parameters during COVID-19 hospitalization. Significant negative correlations are shown in orange and significant positive correlations in blue. Color intensity increases with the magnitude of correlation. White colored cells indicate a non-significant correlation

Follow-up of lipid profile was performed during hospitalization in 1109 patients (56.7% males, mean age 67.8 ± 15.3) who had two or more consecutive determinations of the lipid profile. Total cholesterol (T-c), HDL-c, and LDL-c decreased in 28.7%, 42.9%, and 40.0% of patients, respectively. On the other hand, more than 75% of patients had an increase in TG levels during follow-up (median TG increased from 33 to 127 mg/dl). A decline in T-c was observed during the progression phase of the disease (Fig. 3a). Likewise, a similar trend was observed for HDL-c profile during COVID hospitalization (Fig. 3a). LDL-c did not vary during the phase of progression, except for the patients that were admitted to the ICU, who had a rapid increase in LDL-c during that phase of the disease (Fig. 3a). During hospitalization, TG levels showed a progressive increase and were particularly high in those admitted to the ICU (Fig. 3a).

Regarding the correlation of variation of these lipid profile markers with parameters of inflammation, we found that a variation in TG levels was correlated with ferritin and D-dimer, but not with HsCRP (Fig. 3b). Interestingly, a correlation was found between albumin levels and T-c (*r* = 0.39, *p* < 0.05) as well as between albumin levels and HDL-c (*r* = 0.37, *p* < 0.05) (Fig. 3b). As with HDL-c values, albumin levels were lower in both the non-survivor group and the patients admitted to the ICU during follow-up. TG levels steadily increased during hospitalization and showed a “delayed inflammation pattern” (Fig. 3a) that was similar to ferritin and D-Dimer (Fig. 3c), whereas hsCRP had a different acute inflammation pattern (peak during initial admission) (Fig. 3c). The behavior of hsCRP, ferritin, and D-dimer, according to ICU admission and in-hospital mortality, was similar to previous reports.

### Decrease in total cholesterol and its fractions and increase in TG levels are prognostic factors for disease progression

Disease severity was considered as progression to severe respiratory distress (advanced ventilation maneuvers), critical stage, or death. In the multivariable Cox model for ICU admission prediction (Table 4), higher values of T-c (HR 0.55 95% CI 0.36–0.86 for 50 mg/dL variation), HDL-c (HR 0.61 95% CI 0.45–0.84 for 10 mg/dL variation), and LDL-c showed a protective effect (HR 0.85 95% CI 0.74–0.97 for 10 mg/dL variation) after adjusting for age, gender, comorbidities, and inflammation parameters (hsRCP, D-dimer and ferritin). In addition, in the multivariable Cox model for mortality prediction (Table 4), higher values of T-c (HR 0.45 95% CI 0.36–0.57 for each 50 mg/dL variation), HDL-c (HR 0.66 95% CI 0.54–0.81 for 10 mg/dL variation), and LDL-c (HR 0.86 95% CI 0.78–0.94 for 10 mg/dL variation) also showed a protective effect on mortality after adjusting for age, gender comorbidities, and inflammation parameters (hsRCP, D-dimer, and ferritin). TG were not associated with mortality in the Cox regression analyses (Table 4).

**Table 4:** Cox regression analysis for association with mortality and ICU admission in COVID-19 patients

Lipid profile findings	ICU admission	
	HR^a^	(95% CI)
Pre-ICU T-c (50 mg/dL variation)	0.55	(0.36–0.86)
Pre-ICU HDL-C values (10 mg/dL variation)	0.61	(0.45–0.84)
Pre-ICU LDL-C values (10 mg/dL variation)	0.85	(0.74–0.97)
Pre-ICU TG values (100 mg/dL variation)	1.07	(0.94–1.23)
	**Mortality**	
Max T-c (50 mg/dL variation)	0.45	(0.36–0.57)
Max HDL-C values (10 mg/dL variation)	0.66	(0.54–0.81)
Max LDL-C values (10 mg/dL variation)	0.86	(0.78–0.94)
Max TG values (100 mg/dL variation)	0.94	(0.81–1.09)

^a^Ratios adjusted by age, sex, comorbidities, and inflammation parameters (hsRCP, D-dimer and ferritin)

## Discussion

In our study of hospitalized COVID-19 patients, we found a significant decrease in T-c, LDL-c, and HDL-c levels as well as an increase in TG at hospital admission. Lipid abnormalities have been associated with different infections, especially with sepsis [7–9]. The most widely recognized association of virus infection with dyslipidemia is HIV [10]. HIV-1 infection has been associated with an elevation in TG along with decreased levels of HDL-c [11], a similar pattern to the one found in COVID-19 patients. A potential explanation of dyslipidemia in the HIV infection is that the virus may damage liver cells, consequently altering both TG and cholesterol synthesis [11]. In this regard, we found a correlation between decreased albumin levels and a decrease in T-c and HDL-c, as well as between decreased albumin levels and an increase in TG, pointing out to a dysfunction in protein synthesis at the liver as a possible pathogenic mechanism. Another possible mechanism of dyslipidemia could be the side effects of medications. LPV/r and tocilizumab have been associated with increased TG in SARS-CoV-2 infection [12, 13]. However, in our cohort of patients the only relationship we found was between tocilizumab therapy and the consequent increase in TG levels. Moreover, Tocilizumab had been previously associated with hyperTG in 2 patients who received this therapy for the cytokine storm associated with COVID-19 [13, 14].

The decrease in T-c, LDL-c, and/or HDL-c levels was associated with increased severity and mortality for COVID-19 and was independent of the presence of comorbidities and other known inflammatory biomarkers (hsCRP, D-dimer, and ferritin). However, no effect on severity and mortality was found for TG when adjusted in the multivariable analysis. Previous reports of hospitalized COVID-19 patients have found that low HDL-c levels at admission, and in some cases low LDL-c levels, were predictive of a more severe disease [15–19]. In our patients, we observed that during hospitalization T-c, LDL-c, and HDL-c levels continued to decrease while TG levels increased in those patients with a severe form of the disease. Some similarities regarding follow-up of lipid abnormalities have been previously reported in two series of COVID patients [17, 20]. However, not all reports have found a relationship between T-c, LDL-c and/or TG levels, and the severity of the disease [15, 18, 21]. Interestingly, there have been recent reports that HDL particles may not be only low but also have qualitative abnormalities associated with low ApoA-I levels suggesting that low HDL-c in severe COVID-19 patients might be associated with an important disfunction on endothelial cells towards inflammatory conditions [22].

We also found a clear association of both the elevation of TG levels and the decrease in HDL-c, with markers of inflammation in COVID-19 patients. Interestingly, the correlation was stronger in markers that cause a delayed inflammatory pattern, such as ferritin and D-Dimer, and weaker in markers related with an acute inflammatory pattern, such as hsCRP. HyperTG has been related to inflammation during the systemic inflammatory response syndrome where the TG increase is proportional to different inflammation markers including procalcitonin and pro-inflammatory cytokines [23]. In fact, pro-inflammatory cytokines, particularly IL-6, have been associated with HDL dysfunction [24]. One of the most important mechanisms underlying severity of COVID-19 is the so-called “cytokine storm”, which is characterized by an overproduction of several cytokines that can lead to acute respiratory distress syndrome or even multiple organ failure [25]. COVID-19 could expose patients to remarkably high levels of circulating inflammatory molecules that can induce or even worsen hyperTG, as well as decrease HDL levels. Interestingly, the levels of inflammatory markers are inversely correlated with albumin levels, pointing out to these inflammatory molecules as possible inhibitors of protein synthesis in hepatocytes, which could affect the synthesis of lipoproteins, albumin, and coagulation factors [26]. We have to highlight that lipid profile is a cheap routine test that can be carried out in virtually all clinical settings, and that could also be used as an independent risk factor associated with severity of the inflammatory process in COVID-19 patients.

In addition, an increase in TG levels and a decrease in HDL-c could also have a direct deleterious effect in the endothelium of COVID-19 patients. This atherogenic pattern has been associated with endothelial dysfunction, platelet activation, and coagulation, promoting thrombotic and/or cardiovascular events, as has been reported in diabetic patients [27]. In our cohort of patients, we found a direct relationship between an increase in TG and an increase in D-dimer, as well as a decrease in HDL and an increase in D-dimer, which is a marker of coagulopathy in COVID-19 [28]. This could suggest that these lipid abnormalities may act synergistically with SARS-CoV-2 to facilitate and/or accelerate endothelial dysfunction and/or coagulopathies that are risk factors of severity and fatality in COVID-19 [29]. As hyperlipidemia could be a significant contributor to endothelial dysfunction, lowering TG and augmenting HDL levels with statins or fibrates could protect the endothelial integrity from the SARS-CoV-2 infection.

In this study we did not find that patients under statin therapy had decreased mortality and morbidity during SARS-CoV-2 infection as reported by others [30]. When statin use was studied after a multivariable analysis adjusted by comorbidities, including hypertension, diabetes, cardiovascular and cerebrovascular disease, no differences were found in survival or severity of disease. The role of statins in COVID-19 patients has been controversial on their effect in severity of the disease, they have been found to be mostly beneficial, but in some studies results have been negative or neutral [30].

This study has several limitations. Firstly, it was conducted during a large-scale infectious disease outbreak of COVID-19, when the healthcare system was overwhelmed by the large number of patients being admitted. Although medical care followed a protocol, it was a retrospective study in nature and it took place at a university hospital in Madrid, potentially limiting generalization to other hospital settings. Secondly, the current study only included hospitalized patients. Hence, we need to further study if these findings can be extrapolated to non-hospitalized patients with a milder disease. Thirdly, patients sought medical attention at varying stages in their illness course, which could have affected their clinical course and outcomes. To mitigate the potential bias, we analyzed serial concentrations of these and other biomarkers on the same samples and related extraction date to the date of symptom onset. In addition, as the determination of lipid profile was not usually performed on the day of admission by protocol, some severe patients had died before drawing a blood sample which included the lipid parameters and therefore, could not be included in the study.

In summary, COVID19 infection probably has an impact on lipid metabolism that is further influenced by inflammatory mechanisms and specific therapies. A correlation between these lipid abnormalities and development of severe outcomes was found. Future studies should investigate if a dysfunctional lipid profile could act synergistically with the SARS-CoV-2 infection and, therefore, facilitate and accelerate the development of vasculopathies and or coagulopathies.

## Data Availability

Some or all datasets generated during and/or analyzed during the current study are not publicly available but are available from the corresponding author on reasonable request.
